# Locating the Sets of Exceptional Points in Dissipative Systems and the Self-Stability of Bicycles

**DOI:** 10.3390/e20070502

**Published:** 2018-07-01

**Authors:** Oleg N. Kirillov

**Affiliations:** Northumbria University, Newcastle upon Tyne NE1 8ST, UK; oleg.kirillov@northumbria.ac.uk; Tel.: +44-(0)191-243-7611

**Keywords:** exceptional points in classical systems, coupled systems, non-holonomic constraints, nonconservative forces, stability optimization, spectral abscissa, swallowtail, bicycle self-stability

## Abstract

Sets in the parameter space corresponding to complex exceptional points (EP) have high codimension, and by this reason, they are difficult objects for numerical location. However, complex EPs play an important role in the problems of the stability of dissipative systems, where they are frequently considered as precursors to instability. We propose to locate the set of complex EPs using the fact that the global minimum of the spectral abscissa of a polynomial is attained at the EP of the highest possible order. Applying this approach to the problem of self-stabilization of a bicycle, we find explicitly the EP sets that suggest scaling laws for the design of robust bikes that agree with the design of the known experimental machines.

## 1. Introduction

Exceptional points in classical systems have recently attracted the attention of researchers in the context of the parity-time (PT) symmetry found in mechanics [1,2] and electronics [3]. In the context of the stability of classical systems, the PT-symmetry plays a part in systems of coupled mechanical oscillators with the indefinite matrix of damping forces [4,5,6,7,8]. Stable PT-symmetric indefinitely-damped mechanical systems have imaginary eigenvalues and thus form singularities on the boundary of the domain of the asymptotic stability of general dissipative systems [9,10]. Among these singularities are exceptional points corresponding to double imaginary eigenvalues with the Jordan block. They belong to sets of complex exceptional points with nonzero real parts that live both in the domain of instability and in the domain of asymptotic stability of a dissipative system and pass through the imaginary exceptional points on the stability boundary that bound the region of PT-symmetry [11,12]. These are sets of high codimension, which are hard to find by numerical approaches. Nevertheless, in many applications, it was realized that complex exceptional points hidden inside the domain of asymptotic stability significantly influence the transition to instability [13,14]. How does one locate the set of complex exceptional points? The general approach involving commutators of matrices of the system [15,16] does not look easily interpretable. In this paper, we will use a recent observation [17] that the set of complex exceptional points connects the imaginary exceptional points on the boundary of asymptotic stability and the real exceptional points inside the domain of asymptotic stability that lie on the boundary of the domain of heavy damping. We will show how the location of the exceptional points with this approach helps to find explicit scaling laws in the classical problem of the self-stability of bicycles.

## 2. Complex Exceptional Points and the Self-Stability of Bicycles

The bicycle is easy to ride, but surprisingly difficult to model [18]. Refinement of the mathematical model of a bicycle has continued over the last 150 years with contributions from Rankine, Boussinesq, Whipple, Klein, Sommerfeld, Appel, Synge and many others [19,20,21]. The canonical, nowadays commonly-accepted model goes back to the 1899 work by Whipple. The Whipple bike is a system consisting of four rigid bodies with knife-edge wheels making it non-holonomic, i.e., requiring for its description more configuration coordinates than the number of its admissible velocities [22,23]. Due to the non-holonomic constraints, even the bicycle tire tracks have a non-trivial and beautiful geometry that has deep and unexpected links to integrable systems, particle traps and the Berry phase [24,25,26].

A fundamental empirical property of real bicycles is their self-stability without any control at a sufficiently high speed [27]. This property has a number of important practical implications. For instance, recent experiments confirm the long-standing assumption that the bicycle designs that do not present the self-stability are difficult for a person to ride; in other words, more stable bikes handle better [18,28,29]. Hence, deeper understanding of the passive stabilization can provide new principles for the design of more safe and rideable bicycles, including compact and foldable models. Furthermore, it is expected to play a crucial part in formulating principles of the design of energy-efficient wheeled and bipedal robots [30].

However, the theoretical explanation of the self-stability has been highly debated throughout the history of bicycle dynamics [22] to such an extent that a recent news feature article in Nature described this as “the bicycle problem that nearly broke mathematics” [18]. An excellent scientific and historical review of thoughts on the bicycle self-stability can be found in [21].

The reason as to why “simple questions about self-stabilization of bicycles do not have straightforward answers” [20] lies in the symbolical complexity of the Whipple model that contains seven degrees of freedom and depends on 25 physical and design parameters [19]. In recent numerical simulations [19,20,22], self-stabilization has been observed for some benchmark designs of the Whipple bike. These results suggested further simplification of the model yielding a reduced model of a bicycle with vanishing radii of the wheels (which are replaced by skates; see, e.g., [31]), known as the two-mass-skate (TMS) bicycle [27,29]. Despite the self-stable TMS bike having been successfully realized in recent laboratory experiments [27], its self-stability still awaits a theoretical explanation.

In the following, we will show how the location of complex and real exceptional points allows one to find hidden symmetries in the model, suggesting further reduction of the parameter space and, finally, providing explicit relations between the parameters of stability-optimized TMS bikes.

### 2.1. The TMS Bicycle Model

**Figure 1:**
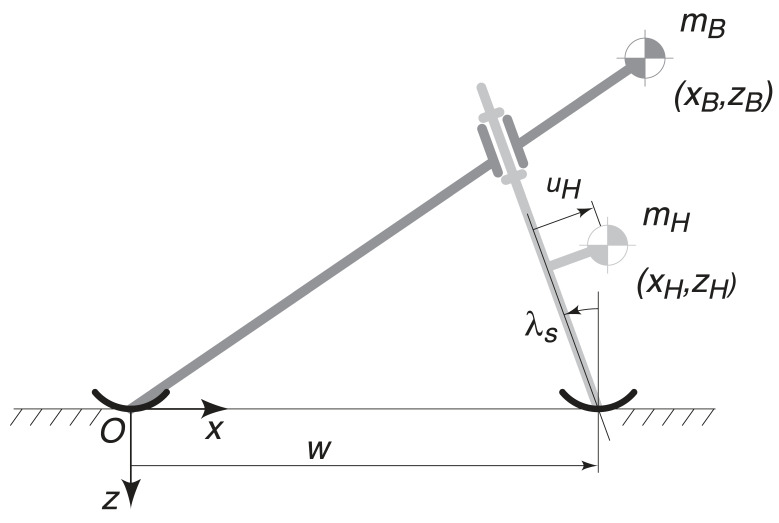
The two-mass-skate (TMS) bicycle model [27].

The TMS model is sketched in Figure 1. It depends on nine dimensional parameters:

$$w,\;v,\;\lambda_{s},\;m_{B},\;x_{B},\;z_{B},\;m_{H},\;x_{H},\;z_{H}$$

that represent, respectively, the wheel base, velocity of the bicycle, steer axis tilt, rear frame assembly (*B*) mass, horizontal and vertical coordinates of the rear frame assembly centre of mass, front fork and handlebar assembly (*H*) mass and horizontal and vertical coordinates of the front fork and handlebar assembly centre of mass [27]; see Table 1.

We wish to study the stability of the TMS bicycle that is moving along a straight horizontal trajectory with a constant velocity and remaining in a straight vertical position. In order to simplify the analysis, it is convenient to choose the wheelbase, *w*, as a unit of length and to introduce the dimensionless time $\tau =t\sqrt{\frac{g}{w}}$ and seven dimensionless parameters:

$$\mathrm{Fr}=\frac{v}{\sqrt{gw}},\;\;\mu =\frac{m_{H}}{m_{B}},\;\;\chi_{B}=\frac{x_{B}}{w},\;\;\chi_{H}=\frac{x_{H}}{w},\;\;\zeta_{B}=\frac{z_{B}}{w},\;\;\zeta_{H}=\frac{z_{H}}{w},\;\;\lambda_{s},$$

where *g* is the gravity acceleration, Fr the Froude number and μ the mass ratio; see Table 1. Notice that $\zeta_{B}\leq 0$ and $\zeta_{H}\leq 0$ due to the choice of the system of coordinates; see Figure 1.

It has been shown in [19,27] that small deviations from the straight vertical equilibrium of the TMS bicycle are described by the leaning angle, ϕ, of the frame and the steering angle, δ, of the front wheel/skate. These angles are governed by the two coupled linear differential equations:

(1) $$M\ddot{q}+V\dot{q}+Pq=0,\;q={\left(\phi ,\delta \right)}^{T},$$

where dot denotes differentiation with respect to dimensionless time, τ, and the matrices of mass, M, velocity-dependent forces, V, and positional forces, P, are:

(2) $$\begin{array}{c} M=\left(\begin{array}{cc} \mu \zeta_{H}^{2}+\zeta_{B}^{2} & -\mu \zeta_{H}\nu_{H}\\ -\mu \zeta_{H}\nu_{H} & \mu \nu_{H}^{2} \end{array}\right),\;V=\left(\begin{array}{cc} 0 & -\mu \chi_{H}\zeta_{H}-\chi_{B}\zeta_{B}\\ 0 & \mu \chi_{H}\nu_{H} \end{array}\right)\mathrm{Fr}\cos\lambda_{s},\\ P=\left(\begin{array}{cc} \mu \zeta_{H}+\zeta_{B} & -{\mathrm{Fr}}^{2}\cos\lambda_{s}\left(\mu \zeta_{H}+\zeta_{B}\right)-\mu \nu_{H}\\ -\mu \nu_{H} & \mu \left({\mathrm{Fr}}^{2}\cos\lambda_{s}-\sin\lambda_{s}\right)\nu_{H} \end{array}\right), \end{array}$$

respectively, with $\nu_{H}=\frac{u_{H}}{w}=\left(\chi_{H}-1\right)\cos\lambda_{s}-\zeta_{H}\sin\lambda_{s}$; see Figure 1.

### 2.2. Preliminaries on Lyapunov Stability and the Asymptotic Stability of Equilibria

An equilibrium of a nonlinear dynamical system is said to be Lyapunov stable if all the solutions starting in its vicinity remain in some neighbourhood of the equilibrium in the course of time [17,32,33]. For asymptotic stability, the solutions are required, additionally, to converge to the equilibrium as time tends to infinity. The first (indirect) method of Lyapunov reduces the study of the asymptotic stability of an autonomous (time-independent) system to the problem of location in the complex plane of eigenvalues of the operator of its linearization [32]. In a finite-dimensional case, the eigenvalues are roots of a polynomial characteristic equation. Localization of all the roots in the open left half of the complex plane is a necessary and sufficient condition for the asymptotic stability of a linearization, which usually implies the asymptotic stability of the original non-linear system [32]. The algebraic Routh–Hurwitz criterion provides explicit conditions for asymptotic stability expressed in terms of the coefficients of the characteristic polynomial [33]. The Lienard–Chipart criterion is an equivalent version of the Routh–Hurwitz criterion, which sometimes gives simpler expressions for the stability conditions [33].

The solution to the linear differential equation is a linear combination of exponential functions with the argument equal to time multiplied by an eigenvalue. Consequently, in the domain of asymptotic stability, solutions of the linearization exponentially decay in time and either have oscillations, which corresponds to a complex eigenvalue with the negative real part, or do not have oscillations, which corresponds to a negative real eigenvalue. If all the solutions exponentially decay without oscillations, i.e., all eigenvalues are real and negative, the system is said to be heavily damped [33,34,35]. A perturbed heavily-damped system quickly and monotonously returns to its equilibrium, which is perceived by an observer as a robust stability. For this reason, placement of the parameters of a system in the domain of heavy damping is a desirable goal in many engineering applications [34,35]. Naturally, heavy damping implies asymptotic stability, and therefore, the domain of heavy damping belongs to the domain of asymptotic stability in the parameter space [17].

Similarly, in the domain of instability, a complex eigenvalue with the positive real part corresponds to an oscillatory solution with the exponentially-growing amplitude. This unstable behaviour is frequently called flutter, dynamic instability, oscillatory instability or Hopf bifurcation in different engineering and physical contexts [33]. In the context of bicycle dynamics, the growing oscillations are referred to as the weaving instability [19,20]. A positive real eigenvalue corresponds to the static instability (or steady-state bifurcation) of an equilibrium described by a non-oscillatory solution with an exponentially-growing amplitude. A bicycle is capsizing in this case [19,20].

With the change of parameters of the system, one can move from the domain of instability to the domain of asymptotic stability in the parameter space. This transition is accompanied by the crossing of the imaginary axis in the complex plane either by at least one pair of complex-conjugate simple eigenvalues or by at least one real eigenvalue. Exactly on the stability boundary, the eigenvalues become imaginary or zero, respectively. In multiple parameter systems, multiple imaginary or zero eigenvalues with different algebraic and geometric multiplicities are generically possible on the stability boundary. In physics, a point in the parameter space corresponding to a linear operator (matrix, matrix polynomial) with the multiple eigenvalues that has less eigenvectors than its algebraic multiplicity (i.e., an operator has a non-trivial Jordan normal form) is called an exceptional point. Frequently, the very multiple eigenvalues with the Jordan block in the complex plane are referred to as exceptional points. Exceptional points form geometric singularities both on the boundary of asymptotic stability and on the boundary of the domain of heavy damping [17,33]. Moreover, exceptional points corresponding to complex eigenvalues exist inside both the domain of asymptotic stability and the domain of instability. Below, we uncover all the exceptional points in the TMS bicycle model and, with their use, find optimal TMS bikes with respect to different stability criteria.

### 2.3. Asymptotic Stability of the TMS Bike and the Critical Froude Number for the Weaving Motion

The TMS model (1), (2) is autonomous and nonconservative, containing dissipative, gyroscopic, potential and non-potential positional (circulatory [33], curl [36]) forces. Assuming the exponential solution ∼exp(sτ) of the linear system (1) and computing $\det(Ms^{2}+Vs+P)$, we write the characteristic polynomial of the TMS bicycle model:

(3) $$p\left(s\right)=a_{0}s^{4}+a_{1}s^{3}+a_{2}s^{2}+a_{3}s+a_{4},$$

with the coefficients:

(4) $$\begin{array}{ccc} a_{0} & = & -\left(\zeta_{H}\tan\lambda_{s}-\chi_{H}+1\right)\zeta_{B}^{2},\\ a_{1} & = & \mathrm{Fr}\left(\zeta_{B}\chi_{H}-\zeta_{H}\chi_{B}\right)\zeta_{B},\\ a_{2} & = & {\mathrm{Fr}}^{2}\left(\zeta_{B}-\zeta_{H}\right)\zeta_{B}-\zeta_{B}\left(\zeta_{B}+\zeta_{H}\right)\tan\lambda_{s}-\left(\chi_{H}-1\right)\left(\mu \zeta_{H}-\zeta_{B}\right),\\ a_{3} & = & -\mathrm{Fr}\left(\chi_{B}-\chi_{H}\right)\zeta_{B},\\ a_{4} & = & -\zeta_{B}\tan\lambda_{s}-\mu \left(\chi_{H}-1\right). \end{array}$$

Applying the Lienard–Chipart version of the Routh–Hurwitz criterion [33,37] to the polynomial (3) yields for $\lambda_{s}>0$ the following necessary and sufficient conditions for the asymptotic stability of the TMS bicycle:

(5) $$\begin{array}{ccc} \chi_{H} & > & 1+\zeta_{H}\tan\lambda_{s},\\ \chi_{H} & < & 1-\frac{\zeta_{B}}{\mu }\tan\lambda_{s},\\ \chi_{H} & < & \chi_{B},\\ \zeta_{H} & > & \zeta_{B},\\ \mathrm{Fr} & > & {\mathrm{Fr}}_{c}>0, \end{array}$$

where the critical Froude number at the stability boundary is given by the expression:

(6) $${\mathrm{Fr}}_{c}^{2}=\frac{\zeta_{B}-\zeta_{H}}{\chi_{B}-\chi_{H}}\frac{\chi_{B}\chi_{H}}{\zeta_{B}\chi_{H}-\zeta_{H}\chi_{B}}\tan\lambda_{s}+\frac{\chi_{H}-1}{\chi_{B}-\chi_{H}}\frac{\chi_{H}}{\zeta_{B}}\mu -\frac{\chi_{H}-1}{\zeta_{B}\chi_{H}-\zeta_{H}\chi_{B}}\chi_{B}.$$

At $0\leq \mathrm{Fr}<{\mathrm{Fr}}_{c}$, the bicycle is unstable, while at $\mathrm{Fr}>{\mathrm{Fr}}_{c}$, it is asymptotically stable. The critical value ${\mathrm{Fr}}_{c}$ is on the boundary between the domains of the asymptotic stability and dynamic instability (weaving motion, [19,20,27]). Notice that in the recent work [38], a comprehensive analysis of the Lienard–Chipart conditions for the TMS bicycle reduced self-stable designs to just two classes corresponding to either positive or negative angles $\lambda_{s}$ and excluded backward stability for the TMS model. Here, we limit our analysis to the $\left(\lambda_{s}>0\right)$-class of the self-stable TMS bikes.

For instance, for the wheel base w=1 m, the design proposed in [27] is determined by:

(7) $$\begin{array}{c} \lambda_{s}=\frac{5\pi }{180}\;\mathrm{rad},\;\;m_{H}=1\;\mathrm{kg},\;\;m_{B}=10\;\mathrm{kg},\;\;x_{B}=1.2\;m,\;\;x_{H}=1.02\;m,\;\;z_{B}=-0.4\;m,\;\;z_{H}=-0.2\;m. \end{array}$$

With (7), we find from (6) the critical Froude number and the corresponding critical velocity:

(8) $${\mathrm{Fr}}_{c}=0.9070641497,\;\;v_{c}=2.841008324\;m/s$$

that reproduce the original result obtained numerically in [27].

### 2.4. Minimizing the Spectral Abscissa of General TMS Bikes

The criterion (5) guarantees the asymptotic stability of the bicycle at $\mathrm{Fr}>{\mathrm{Fr}}_{c}$. However, the character of the time dependence of the steering and leaning angles could be different at different points within the domain of asymptotic stability. Indeed, complex eigenvalues with negative real parts correspond to exponentially-decaying oscillatory motions, whereas negative real eigenvalues yield the exponential decay of perturbations without oscillations. Recall that if all the eigenvalues of the system are real and negative, the system is heavily damped [34,35]. If we wish that the deviations from the straight vertical position of the heavily-damped TMS bike riding along a straight line also quickly die out, we need to maximize the decay rates of the deviations in the following sense.

The abscissa of the polynomial p(s) is the maximal real part of its roots:

a(p)=maxRes:p(s)=0.

Minimization of the spectral abscissa over the coefficients of the polynomial provides a polynomial with the roots that have minimal possible real parts (maximal possible decay rates). In the case of the system of coupled oscillators of the form (1), it is known that the global minimum of the spectral abscissa is $a_{min}=\omega_{0}$, where $\omega_{0}=-\sqrt[{4}]{\frac{\det P}{\det M}}$ [39,40]. Knowing the coefficients of the characteristic polynomial (4), it is easy to find that for the TMS bicycle:

(9) $$\omega_{0}=-\sqrt[{4}]{\frac{1}{\zeta_{B}^{2}}\frac{\zeta_{B}\tan\lambda_{s}+\mu \left(\chi_{H}-1\right)}{\zeta_{H}\tan\lambda_{s}-\left(\chi_{H}-1\right)}}.$$

Remarkably, if $s=\omega_{0}$ is the minimum of the spectral abscissa, it is the four-fold root of the fourth-order characteristic polynomial (3), which is the quadruple negative real eigenvalue with the Jordan block of order four of the linear operator $Ms^{2}+Vs+P$ [17,39]. In this case, the polynomial (3) takes the form:

(10) $$p\left(s\right)={\left(s-\omega_{0}\right)}^{4}=s^{4}-4s^{3}\omega_{0}+6s^{2}\omega_{0}^{2}-4s\omega_{0}^{3}+\omega_{0}^{4},\;\omega_{0}^{4}=\frac{a_{4}}{a_{0}}=\frac{\det P}{\det M}.$$

Comparing (3) and (10), we require that:

$$\begin{array}{ccc} a_{1} & = & \mathrm{Fr}\left(\zeta_{B}\chi_{H}-\zeta_{H}\chi_{B}\right)\zeta_{B}=-4\omega_{0}a_{0},\\ a_{3} & = & -\mathrm{Fr}\left(\chi_{B}-\chi_{H}\right)\zeta_{B}=-4\omega_{0}^{3}a_{0}. \end{array}$$

Dividing the first equation by the second one, we get the relation:

$$\frac{\zeta_{B}\chi_{H}-\zeta_{H}\chi_{B}}{\chi_{B}-\chi_{H}}=\frac{-1}{\omega_{0}^{2}}$$

that we resolve with respect to $\chi_{B}$ to obtain the following design constraint (or scaling law):

(11) $$\chi_{B}=\frac{\omega_{0}^{2}\zeta_{B}-1}{\omega_{0}^{2}\zeta_{H}-1}\chi_{H}.$$

Another constraint follows from the requirement $a_{2}=6\omega_{0}^{2}a_{0}$:

(12) $${\mathrm{Fr}}^{2}\left(\zeta_{B}-\zeta_{H}\right)+\left(6\omega_{0}^{2}\zeta_{H}\zeta_{B}-\zeta_{B}-\zeta_{H}\right)\tan\lambda_{s}=\zeta_{B}^{-1}\left(\chi_{H}-1\right)\left(6\omega_{0}^{2}\zeta_{B}^{2}+\mu \zeta_{H}-\zeta_{B}\right).$$

Let us optimize the stability of the benchmark (7). Set, for example, $\omega_{0}=-1$. Then, taking from the benchmark (7) the parameters $\zeta_{B}=-0.4$, $\zeta_{H}=-0.2$ and $\chi_{H}=1.02$, we find from Equation (11) that $\chi_{B}=1.19$. With these values, the constraint (12) is:

(13) $$-0.432\tan\lambda_{s}-0.0272+0.08{\mathrm{Fr}}^{2}+0.004\mu =0,$$

the relation (9) yields:

(14) $$0.368\tan\lambda_{s}-0.02\mu -0.0032=0,$$

and the characteristic polynomial evaluated at s=−1 results in the equation:

(15) $$0.192\tan\lambda_{s}-0.0048-0.136\mathrm{Fr}+0.08{\mathrm{Fr}}^{2}-0.016\mu =0.$$

The system (13)–(15) has a unique solution with the mass ratio μ>0:

$$\mathrm{Fr}=2.337214017,\;\mu =20.84626701,\;\lambda_{s}=0.8514403685.$$

**Figure 2:**
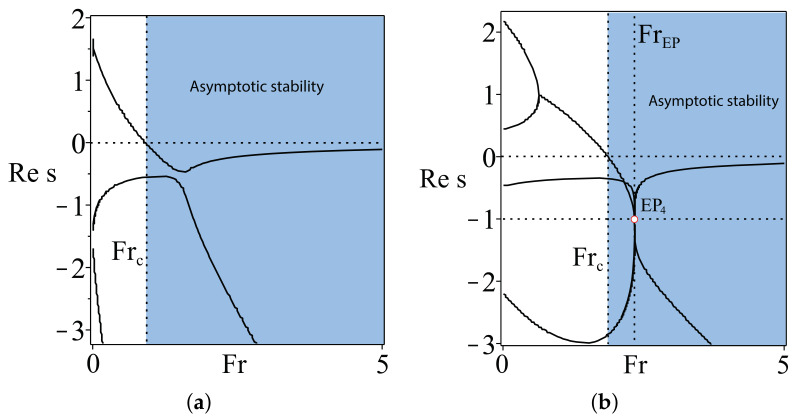
(**a**) The growth rates for the benchmark TMS bicycle (7); (**b**) the growth rates of the optimized TMS bicycle with $\zeta_{B}=-0.4$, $\zeta_{H}=-0.2$, $\chi_{B}=1.19$, $\chi_{H}=1.02$, μ=20.84626701 and $\lambda_{s}=0.8514403685$ showing that the spectral abscissa attains its minimal value $a_{min}=-1$ at ${\mathrm{Fr}}_{\mathrm{EP}}=2.337214017$ at the real exceptional point of order four, ${\mathrm{EP}}_{4}$.

This means that the optimized TMS bicycle attains the global minimum of the spectral abscissa at ${\mathrm{Fr}}_{EP}=2.337214017$ where all four eigenvalues merge into a quadruple negative real eigenvalue s=−1 with the Jordan block, Figure 2b. This eigenvalue we call a real exceptional point of order four and denote as ${\mathrm{EP}}_{4}$. For comparison, we show in Figure 2a the growth rates of a generic benchmark TMS bicycle (7).

Why is the location of the real ${\mathrm{EP}}_{4}$ important? In [17], it was shown that this exceptional point is a swallowtail singularity on the boundary of the domain of heavy damping inside the domain of asymptotic stability of a system with two degrees of freedom. Furthermore, the global minimum of the spectral abscissa occurs exactly at the swallowtail degeneracy. In [17], it was shown that the ${\mathrm{EP}}_{4}$ ‘organizes’ the asymptotic stability, and its knowledge helps to locate other exceptional points governing stability exchange between modes of a coupled system. Below, we demonstrate this explicitly for the TMS bikes with $\chi_{H}=1$.

### 2.5. Self-Stable and Heavily-Damped TMS Bikes with $\chi_{H}=1$

#### 2.5.1. The Critical Froude Number and Its Minimum

Why does $\chi_{H}=1$? First, both the benchmarks reported in [27] and their experimental realizations have $\chi_{H}\approx 1$. Second, this choice leads to a dramatic simplification without affecting the generality of our consideration. Indeed, Expression (6) for the critical Froude number evaluated at $\chi_{H}=1$ reduces to:

(16) $${\mathrm{Fr}}_{c}^{2}=\frac{\zeta_{B}-\zeta_{H}}{\zeta_{B}-\chi_{B}\zeta_{H}}\frac{\chi_{B}}{\chi_{B}-1}\tan\lambda_{s}.$$

Choosing $\chi_{H}=1$ automatically makes ${\mathrm{Fr}}_{c}$ and the stability conditions (5) independent of the mass ratio μ. Additionally, the criteria (5) imply $\chi_{B}>1$ and $|\zeta_{B}\left|>\right|\zeta_{H}|$.

Therefore, choosing $\chi_{H}=1$ reduces the dimension of the parameter space from seven to five. The self-stability of the ($\chi_{H}=1$)–bike depends just on Fr, $\chi_{B}$, $\zeta_{H}$, $\zeta_{B}$ and $\lambda_{s}$.

Given $\zeta_{H}$, $\zeta_{B}$ and $\lambda_{s}$, find the minimum of the critical Froude number (16) as a function of $\chi_{B}$. It is easy to see that the minimum is attained at:

(17) $$\chi_{B}=\sqrt{\frac{\zeta_{B}}{\zeta_{H}}}$$

and its value is equal to:

(18) $${\mathrm{Fr}}_{min}=\sqrt{\frac{\sqrt{|\zeta_{B}|}+\sqrt{|\zeta_{H}|}}{\sqrt{|\zeta_{B}|}-\sqrt{|\zeta_{H}|}}\tan\lambda_{s}}.$$

These results suggest that all the critical parameters for the ($\chi_{H}=1$)-bike can be expressed in a similar elegant manner by means of $\zeta_{H}$, $\zeta_{B}$ and $\lambda_{s}$ only. Let us check these expectations calculating the location of the real exceptional point ${\mathrm{EP}}_{4}$ for the ($\chi_{H}=1$)-bike.

#### 2.5.2. Exact Location of the Real Exceptional Point ${\mathrm{EP}}_{4}$

Indeed, with $\chi_{H}=1$, Expression (9) for the real negative quadruple eigenvalue at ${\mathrm{EP}}_{4}$ yields:

(19) $$\omega_{0}=-\sqrt[{4}]{\frac{1}{\zeta_{B}\zeta_{H}}}.$$

The design constraint (11) reduces to the scaling law:

(20) $$\chi_{B}=\sqrt{\frac{\zeta_{B}}{\zeta_{H}}}$$

which is nothing else but the minimizer (17) of the critical Froude number! Simultaneously solving Equation (12) and the equation $p(\omega_{0})=0$, we find explicitly the second design constraint that determines $\tan\lambda_{s}$ at ${\mathrm{EP}}_{4}$:

(21) $$\tan\lambda_{s}=\frac{\omega_{0}^{2}\left(\zeta_{B}-\zeta_{H}\right)}{16\zeta_{H}}\frac{\left(\zeta_{B}+\zeta_{H}\right)\omega_{0}^{2}-6}{\left(\zeta_{B}+\zeta_{H}\right)\omega_{0}^{2}-2}.$$

Finally, from the same system of equations, we find that the Froude number at ${\mathrm{EP}}_{4}$, ${\mathrm{Fr}}_{{\mathrm{EP}}_{4}}$, is a root of the quadratic equation:

(22) $$\left(\omega_{0}^{2}\zeta_{B}-1\right){\mathrm{Fr}}_{{\mathrm{EP}}_{4}}^{2}+2\omega_{0}^{3}\zeta_{B}{\mathrm{Fr}}_{{\mathrm{EP}}_{4}}-\left(\omega_{0}^{2}\zeta_{B}+1\right)\tan\lambda_{s}=0,$$

where $\omega_{0}$ is given by Equation (19) and $\tan\lambda_{s}$ by Equation (21).

Let us take $\zeta_{H}=-0.2$ and $\zeta_{B}=-0.4$ as in the benchmark (7). Then, (20)–(22) locate the ${\mathrm{EP}}_{4}$ in the space of the parameters, giving (Table 2):

$$\chi_{B}=\sqrt{2},\;\tan\lambda_{s}=\frac{15}{4}-\frac{75}{32}\sqrt{2},\;{\mathrm{Fr}}_{{\mathrm{EP}}_{4}}=\frac{3\sqrt{110\sqrt{2}-120}}{8}\approx 2.236317517.$$

#### 2.5.3. Discriminant Surface and the EP-Set

**Figure 3:**
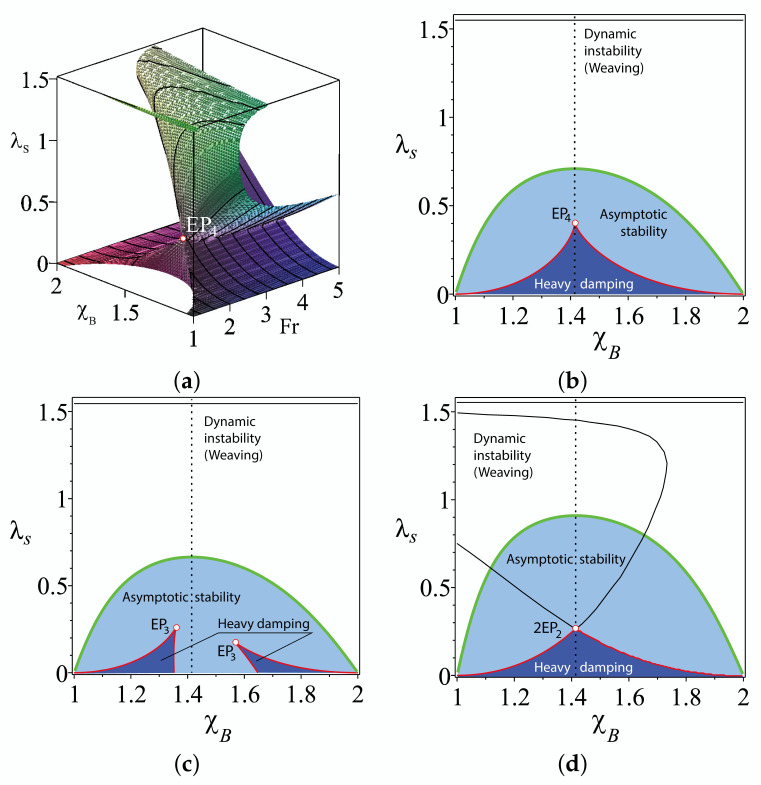
(**a**) The discriminant surface of the characteristic polynomial of the TMS bike with $\chi_{H}=1$, $\zeta_{H}=-0.2$ and $\zeta_{B}=-0.4$ showing the swallowtail singularity at ${\mathrm{EP}}_{4}$. The cross-section of the domain of asymptotic stability and the discriminant surface at (**b**) $\mathrm{Fr}={\mathrm{Fr}}_{{\mathrm{EP}}_{4}}=\frac{3\sqrt{110\sqrt{2}-120}}{8}$, (**c**) $\mathrm{Fr}={\mathrm{Fr}}_{{\mathrm{EP}}_{4}}-0.1$ and (**d**) $\mathrm{Fr}={\mathrm{Fr}}_{{\mathrm{EP}}_{4}}+0.5$.

The located ${\mathrm{EP}}_{4}$ corresponds to a quadruple negative real eigenvalue $s=\omega_{0}=-\frac{\sqrt{5}}{\sqrt[{4}]{2}}$. It is known that ${\mathrm{EP}}_{4}$ is the swallowtail singular point on the discriminant surface of the fourth-order characteristic polynomial [17]. In Figure 3, the discriminant surface is plotted in the $\left(\mathrm{Fr},\chi_{B},\lambda_{s}\right)$-space for the TMS bike with $\chi_{H}=1$, $\zeta_{H}=-0.2$ and $\zeta_{B}=-0.4$ showing the swallowtail singular point with the position specified by the first line of Table 2. The discriminant surface has two cuspidal edges, as well as the line of self-intersection branching from the ${\mathrm{EP}}_{4}$. These singularities belong to the boundary of a domain with the shape of a trihedral spire. This is the domain of heavy damping. In its inner points, all the eigenvalues are real and negative [17].

We see that the line of self-intersection lies in the plane $\chi_{B}=\sqrt{\frac{\zeta_{B}}{\zeta_{H}}}$. Restricted to this plane (parameterized by Fr and $\lambda_{s}$), the discriminant of the characteristic polynomial (3) simplifies and provides the following expression for the curve that contains the line of self-intersection of the discriminant surface:

(23) $$\mathrm{Fr}=\frac{\omega_{0}^{2}\zeta_{B}-1}{\omega_{0}^{2}\zeta_{B}+1}\frac{2\tan\lambda_{s}}{\sqrt{\omega_{0}^{4}\zeta_{B}+4\tan\lambda_{s}\frac{\omega_{0}^{2}\zeta_{B}-1}{\omega_{0}^{2}\zeta_{B}+1}}}.$$

**Figure 4:**
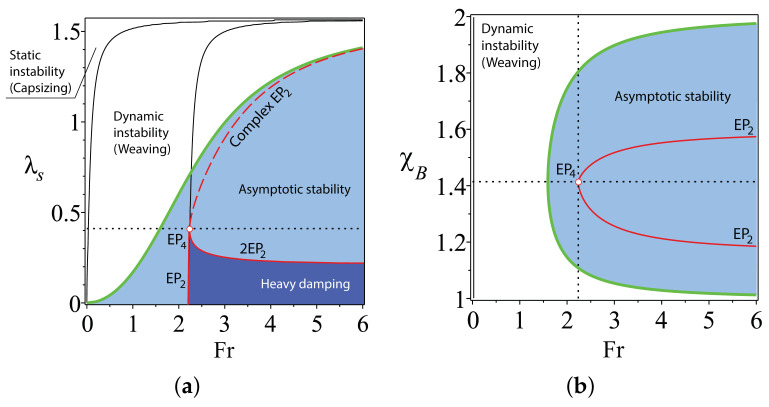
$\chi_{H}=1$, $\zeta_{H}=-0.2$ and $\zeta_{B}=-0.4$. (**a**) For $\chi_{B}=\sqrt{2}$, the boundary between the domains of weaving and asymptotic stability in the $\left(\mathrm{Fr},\lambda_{s}\right)$-plane shown together with the domain of heavy damping that has a cuspidal point corresponding to a negative real eigenvalue $\omega_{0}=-\sqrt[{4}]{\frac{25}{2}}$ with the Jordan block of order four (${\mathrm{EP}}_{4}$). The ${\mathrm{EP}}_{4}$ belongs to a curve (23) that corresponds to (dashed part) conjugate pairs of double complex eigenvalues with the Jordan block of order two (complex ${\mathrm{EP}}_{2}$) and (solid part) to couples of double real negative eigenvalues with the Jordan block of order two ($2{\mathrm{EP}}_{2}$). (**b**) The same in the $\left(\mathrm{Fr},\chi_{B}\right)$-plane at $\lambda_{s}=\arctan\left(\frac{15}{4}-\frac{75}{32}\sqrt{2}\right)$ rad. The domain of heavy damping degenerates into a singular point: the swallowtail singularity.

In Figure 4a, the curve (23) is plotted for $\chi_{H}=1$, $\zeta_{H}=-0.2$, $\zeta_{B}=-0.4$ and $\chi_{B}=\sqrt{2}$ in the $\left(\mathrm{Fr},\lambda_{s}\right)$-plane. A point where this curve has a vertical tangent is the swallowtail singularity or ${\mathrm{EP}}_{4}$. The part of the curve below the ${\mathrm{EP}}_{4}$ is a line of self-intersection of the discriminant surface corresponding to a pair of different negative double real eigenvalues with the Jordan block, i.e., to a couple of real exceptional points, which we denote as ${2\mathrm{EP}}_{2}$.

The curve (23) continues, however, also above the ${\mathrm{EP}}_{4}$. This part, shown by a dashed line in Figure 4a, is the set corresponding to conjugate pairs of complex double eigenvalues with the Jordan block, or complex exceptional points that we denote as ${\mathrm{CEP}}_{2}$. Since the curve (23) is a location of three types of exceptional points; we call it the EP-set. Notice that the codimension of the EP-set is two, and for this reason, its location by numerical approaches is very non-trivial.

#### 2.5.4. Location of the EP-Set and Stability Optimization

What does the location of the EP-set mean for the stability of the TMS bike? Drawing the domain of asymptotic stability together with the discriminant surface and the EP-set in the same plot, we see that the EP-set lies entirely in the domain of asymptotic stability; see Figure 4. The ${2\mathrm{EP}}_{2}$ part of the EP-set bounds the domain of heavy damping in the plane $\chi_{B}=\sqrt{\frac{\zeta_{B}}{\zeta_{H}}}=\sqrt{2}$.

Look now at the cross-sections of the asymptotic stability domain and the discriminant surface in the $\left(\chi_{B},\lambda_{s}\right)$-plane; see Figure 3. Remarkably, the value $\chi_{B}=\sqrt{\frac{\zeta_{B}}{\zeta_{H}}}=\sqrt{2}$ is a maximizer of the steer axis tilt $\lambda_{s}$ both at the onset of the weaving instability and at the boundary of the domain of heavy damping. In the latter case, the maximum is always attained at a singular point in the EP-set: either at ${\mathrm{EP}}_{4}$ when $\mathrm{Fr}={\mathrm{Fr}}_{{\mathrm{EP}}_{4}}$ or at $2{\mathrm{EP}}_{2}$ when $\mathrm{Fr}>{\mathrm{Fr}}_{{\mathrm{EP}}_{4}}$. The global maximum of the steer axis tilt on the boundary of the domain of heavy damping is attained exactly at ${\mathrm{EP}}_{4}$, which is also the point where the spectral abscissa attains its global minimum. Taking into account that $\chi_{B}=\sqrt{\frac{\zeta_{B}}{\zeta_{H}}}=\sqrt{2}$ is a minimizer of the critical Froude number that is necessary for asymptotic stability, we conclude that both of the design constraints, (20) and (21), play a crucial part in the self-stability phenomenon:

*The most efficient self-stable TMS bikes are those that have a better chance to operate in the heavy damping domain and simultaneously have the minimal possible spectral abscissa. In the case when*$\chi_{H}=1$, *these bikes should necessarily follow the scaling laws:*

(24) $$\chi_{B}=\sqrt{\frac{\zeta_{B}}{\zeta_{H}}}\;and\;0<\tan\lambda_{s}\leq \frac{\omega_{0}^{2}\left(\zeta_{B}-\zeta_{H}\right)}{16\zeta_{H}}\frac{\left(\zeta_{B}+\zeta_{H}\right)\omega_{0}^{2}-6}{\left(\zeta_{B}+\zeta_{H}\right)\omega_{0}^{2}-2},\;where\;\omega_{0}=-\sqrt[{4}]{\frac{1}{\zeta_{B}\zeta_{H}}}.$$

**Figure 5:**
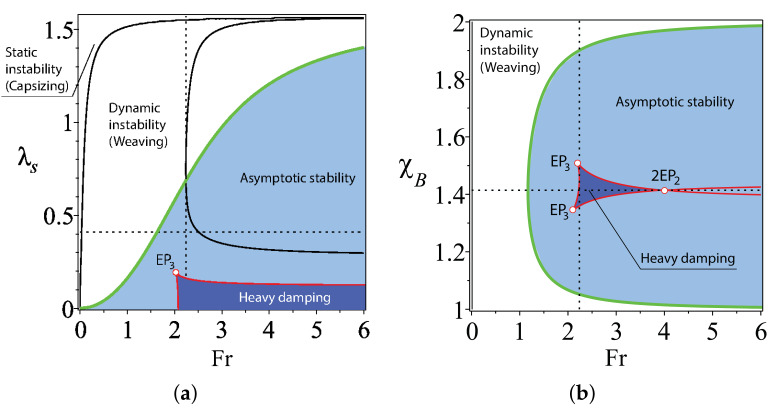
$\chi_{H}=1$, $\zeta_{H}=-0.2$ and $\zeta_{B}=-0.4$. (**a**) For $\chi_{B}=\sqrt{2}-0.1$, the boundary between the domains of weaving and asymptotic stability in the $\left(\mathrm{Fr},\lambda_{s}\right)$-plane shown together with the domain of heavy damping that has a cusp corresponding to a negative real eigenvalue with the Jordan block of order three (${\mathrm{EP}}_{3}$). The ${\mathrm{EP}}_{3}$ belongs to the cuspidal edge of the swallowtail surface bounding the domain of heavy damping. (**b**) The same in the $\left(\mathrm{Fr},\chi_{B}\right)$-plane at $\lambda_{s}=\arctan\left(\frac{15}{4}-\frac{75}{32}\sqrt{2}\right)-0.18$ rad. Notice the cuspidal ${\mathrm{EP}}_{3}$-points and the self intersection at the $2{\mathrm{EP}}_{2}$ point on the boundary of the domain of heavy damping.

Even in the case of an approximate scaling law $\chi_{B}\approx \sqrt{\frac{\zeta_{B}}{\zeta_{H}}}$, the domain of heavy damping is large enough (see Figure 5), suggesting that the formulated principle produces a sufficiently robust design of self-stable TMS bikes.

#### 2.5.5. Mechanism of Self-Stability and ${\mathrm{CEP}}_{2}$ as a Precursor to Bike Weaving

**Figure 6:**
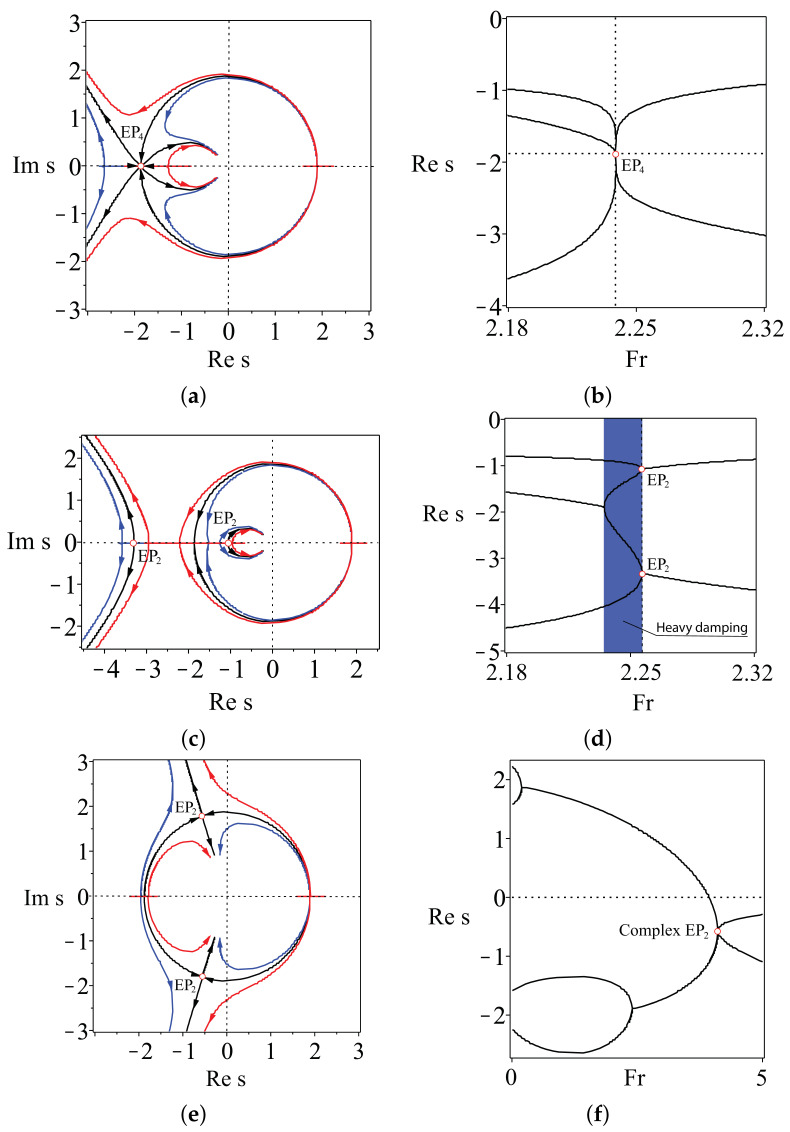
$\chi_{H}=1$, $\zeta_{H}=-0.2$, $\zeta_{B}=-0.4$. Stabilization of the TMS bike as Fr is increasing from 0–5 for (**a,b**) $\lambda_{s}=\arctan\left(\frac{15}{4}-\frac{75}{32}\sqrt{2}\right)$ rad, (**c,d**) $\lambda_{s}=\arctan\left(\frac{15}{4}-\frac{75}{32}\sqrt{2}\right)-0.05$ rad and (**e,f**) $\lambda_{s}=\arctan\left(\frac{15}{4}-\frac{75}{32}\sqrt{2}\right)+0.8$ rad. The eigenvalue curves are shown for (black) $\chi_{B}=\sqrt{2}$, (blue) $\chi_{B}=\sqrt{2}-0.01$ and (red) $\chi_{B}=\sqrt{2}+0.01$ in the upper and middle rows and for (black) $\chi_{B}=\sqrt{2}$, (blue) $\chi_{B}=\sqrt{2}-0.1$ and (red) $\chi_{B}=\sqrt{2}+0.1$ in the lower row. Notice the existence at $\chi_{B}=\sqrt{2}$ of (**a,b**) a real exceptional point ${\mathrm{EP}}_{4}$, (**c,d**) a couple of real exceptional points ${\mathrm{EP}}_{2}$ and (**e,f**) a couple of complex exceptional points ${\mathrm{EP}}_{2}$ and repelling of eigenvalue curves near EPs when $\chi_{B}\neq \sqrt{2}$.

What happens with the stability of TMS bicycles that have a large steer axis tilt? To answer this question, let us look at the movement of eigenvalues in the complex plane at different $\lambda_{s}$ and $\chi_{B}$ as the Froude number increases from 0–5; see Figure 6. At Fr=0, the bicycle is effectively an inverted pendulum, which is statically unstable (capsizing instability [20]) with two real negative eigenvalues and two real positive eigenvalues. As Fr increases, the positive eigenvalues move towards each other along the real axis. The same happens (at a slower rate) with the negative eigenvalues. Eventually, the positive real eigenvalues merge into a double real eigenvalue $s=-\omega_{0}>0$. The subsequent evolution of eigenvalues depends on $\chi_{B}$ and $\lambda_{s}$.

If $\chi_{B}=\sqrt{\frac{\zeta_{B}}{\zeta_{H}}}=\sqrt{2}$, then with the further increase in Fr, the double eigenvalue $s=-\omega_{0}>0$ splits into a conjugate pair of complex eigenvalues with positive real parts, causing weaving instability. This pair evolves along a circle (Res)2+(Ims)2=ω02 and crosses the imaginary axis exactly at $\mathrm{Fr}={\mathrm{Fr}}_{c}$ given by Equation (16), which yields the asymptotic stability of the bicycle.

The further evolution of the eigenvalues depends on the steer axis tilt $\lambda_{s}$; see Figure 6. If $\lambda_{s}$ satisfies the constraint (21), then the complex eigenvalues with the negative real parts moving along the circle approach the real axis and meet the two negative real eigenvalues exactly at $\mathrm{Fr}={\mathrm{Fr}}_{{\mathrm{EP}}_{4}}$, forming a quadruple negative real eigenvalue $s=\omega_{0}$, i.e., the real exceptional point ${\mathrm{EP}}_{4}$. At this moment, all four eigenvalues are shifted as far as possible to the left from the imaginary axis, which corresponds to the global minimum of the spectral abscissa; see Figure 6a,b. A further increase of Fr leads to the splitting of the multiple eigenvalue into a quadruplet of complex eigenvalues with negative real parts (decaying oscillatory motion) and to the increase in the spectral abscissa.

If $\chi_{B}=\sqrt{\frac{\zeta_{B}}{\zeta_{H}}}=\sqrt{2}$ and $\lambda_{s}$ is smaller than the value specified by (21), then the pair moving along the circle reaches the real axis faster than the negative real eigenvalues meet each other; see Figure 6c,d. Then, the complex eigenvalues merge into a double negative real eigenvalue $s=\omega_{0}$, which splits into two negative real ones that move along the real axis in the opposite directions. At these values of Fr, the system has four simple negative real eigenvalues, which correspond to heavy damping. The time evolution of all perturbations is then the monotonic exponential decay, which is favourable for the bike robustness. At $\mathrm{Fr}={\mathrm{Fr}}_{EP}$, which is determined by Equation (23), two real negative double eigenvalues originate simultaneously, marking the formation of the ${2\mathrm{EP}}_{2}$ singularity on the boundary of the domain of heavy damping. A further increase in Fr yields splitting of the multiple eigenvalues into two pairs of complex eigenvalues with negative real parts (decaying oscillatory motion).

If $\chi_{B}=\sqrt{\frac{\zeta_{B}}{\zeta_{H}}}=\sqrt{2}$ and $\lambda_{s}$ is larger than the value specified by (21), then the pair moving along the circle does it so slowly that the real negative eigenvalues manage to merge into a double negative real eigenvalue $s=\omega_{0}$ and then become a pair of two complex eigenvalues evolving along the same circle towards the imaginary axis; see Figure 6e,f. The pairs of complex eigenvalues meet on the circle at $\mathrm{Fr}={\mathrm{Fr}}_{\mathrm{EP}}$, which is determined by Equation (23), i.e., at a point of the EP-set corresponding to a pair of complex exceptional points ${\mathrm{EP}}_{2}$. After the collision, the eigenvalues split into four complex eigenvalues with the negative real parts.

From this analysis, we see that $\lambda_{s}$ indeed determines the balance of the rate of stabilization of unstable modes and the rate of destabilization of stable modes. The former is larger when $\lambda_{s}$ is smaller than the value specified by (21), and the latter is larger when $\lambda_{s}$ exceeds the value specified by (21), thus confirming the design principle (24). The perfect balance corresponds to the angle $\lambda_{s}$ specified by (21), which yields global minimization of the spectral abscissa.

When $\chi_{B}\neq \sqrt{\frac{\zeta_{B}}{\zeta_{H}}}$, then the eigenvalues evolve close to the circle (Res)2+(Ims)2=ω02, but this evolution again differs for different values of $\lambda_{s}$. If for $\lambda_{s}$ smaller than the value specified by (21) the eigenvalue evolution remains qualitatively the same, as is evident from Figure 6c,d, for $\lambda_{s}$ larger than the value specified by (21), the eigenvalues experience strong repulsion near the location of ${\mathrm{CEP}}_{2}$, i.e., when the parameters evolve close to the EP-set of complex exceptional points. Such behaviour of eigenvalues in dissipative systems permanently intrigues many researchers. For instance, Jones [13] remarked in the context of the stability of the plane Poiseuille flow that “unfortunately, it is quite common for an eigenvalue which is moving steadily towards a positive growth rate to suffer a sudden change of direction and subsequently fail to become unstable; similarly, it happens that modes which initially become more stable as [the Reynolds number] increases change direction and subsequently achieve instability. *It is believed that these changes of direction are due to the nearby presence of multiple-eigenvalue points.*” This “nearby presence” of complex exceptional points is elusive unless we manage to locate the EP-set. For the TMS bike, we have obtained this set in the explicit form given by Equations (19), (20) and (23). Dobson et al. [14] posed a question “is strong modal resonance a precursor to [oscillatory instability]?” The strong modal resonance is exactly the interaction of eigenvalues at ${\mathrm{CEP}}_{2}$ shown in Figure 6e,f. Knowing the exact location of the EP-set of complex exceptional points, we can answer affirmatively the question of Dobson et al. Indeed, the complex EP-set shown as a dashed curve in Figure 4a tends to the boundary of asymptotic stability as $\lambda_{s}\to \frac{\pi }{2}$. This means that the ${\mathrm{CEP}}_{2}$ in Figure 6e,f come closer to the imaginary axis at large $\lambda_{s}$, and even small perturbations in $\chi_{B}$ can turn the motion of eigenvalues back to the right-hand side of the complex plane and destabilize the system. Figure 6e,f also demonstrates the selective role of the scaling law $\chi_{B}=\sqrt{\frac{\zeta_{B}}{\zeta_{H}}}$ in determining which mode becomes unstable. The conditions $\chi_{B}>\sqrt{\frac{\zeta_{B}}{\zeta_{H}}}$ and $\chi_{B}<\sqrt{\frac{\zeta_{B}}{\zeta_{H}}}$ affect modes with higher or lower frequency, respectively. In fact, in the limit $\lambda_{s}\to \frac{\pi }{2}$, the dissipative system comes close to a system with a Hamiltonian symmetry of the spectrum. This could be a reversible, Hamiltonian or PT-symmetric system [9,10,12,33], which is very sensitive to perturbations destroying the fundamental symmetry and therefore can easily be destabilized.

#### 2.5.6. How the Scaling Laws Found Match the Experimental TMS Bike Realization

**Figure 7:**
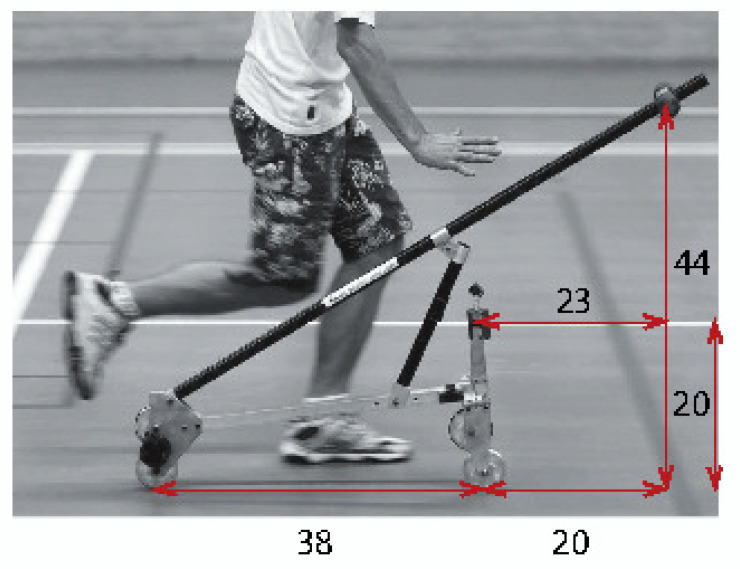
Experimental realization of a self-stable TMS bicycle design found by trial and error in [18,27] with $\chi_{B}=1.526$, $\chi_{H}=0.921$, $\zeta_{B}=-1.158$ and $\zeta_{H}=-0.526$ approximately fitting the scaling law (20). Indeed, $\sqrt{\frac{\zeta_{B}}{\zeta_{H}}}=1.484$ is close to $\chi_{B}=1.526$.

In Figure 7, we show a photograph of the experimental TMS bike from the works [18,27]. If we measure the lengths of the bike from the photo, we can deduce that for this realization, the design parameters are $\chi_{B}=1.526$, $\chi_{H}=0.921$, $\zeta_{B}=-1.158$, $\zeta_{H}=-0.526$. Hence,

$$\sqrt{\frac{\zeta_{B}}{\zeta_{H}}}=1.484\approx \chi_{B}=1.526,$$

which means that the scaling law (20) is matched pretty well. This leads us to the conclusion that the trial-and-error engineering approach to the design of a self-stable TMS bike reported in [27] has eventually produced the design that is close to the optimally-stable one with respect to at least three different criteria: minimization of the spectral abscissa, minimization of ${\mathrm{Fr}}_{c}$ and maximization of the domain of heavy damping. Indeed, our scaling laws (20) and (21) directly follow from the exact optimal solutions to these problems.

## 3. Conclusions

We have found new scaling laws for the two-mass-skate (TMS) bicycle that lead to the design of self-stable machines. These scaling laws optimize the stability of the bicycle by several different criteria simultaneously. The matching of the theoretical scaling laws to the parameters of the TMS bike’s realization demonstrates that the trial-and-error engineering of the bikes selects the most robustly stable species and thus empirically optimizes the bike stability. We have found the optimal solutions directly from the analysis of the sets of exceptional points of the TMS bike model with the help of a general result on the global minimization of the spectral abscissa at an exceptional point of the highest possible order. We stress that all previous results on the self-stability of bicycles even in the linear case have been obtained numerically.

## Figures and Tables

**Table 1:** Notation for the TMS bicycle model.

Dimensional	Meaning	Dimensionless	Meaning
*v*	Velocity of the bike		
*g*	Gravity acceleration	Fr	Froude number
*w*	Wheel base		
$\lambda_{s}$	Steer axis tilt (rad)	$\lambda_{s}$	Steer axis tilt (rad)
$m_{H}$	Front fork and handlebar		
	assembly (FHA) mass	μ	Mass ratio ($m_{H}/m_{B}$)
$m_{B}$	Rear frame assembly (RFA) mass		
$x_{H}$ (≥0)	Horizontal coordinate of the	$\chi_{H}$ (≥0)	Horizontal coordinate of the
	FHA centre of mass		FHA centre of mass
$z_{H}$ (≤0)	Vertical coordinate of the	$\zeta_{H}$ (≤0)	Vertical coordinate of the
	FHA centre of mass		FHA centre of mass
$x_{B}$ (≥0)	Horizontal coordinate of the	$\chi_{B}$ (≥0)	Horizontal coordinate of the
	RFA centre of mass		RFA centre of mass
$z_{B}$ (≤0)	Vertical coordinate of the	$\zeta_{B}$ (≤0)	Vertical coordinate of the
	RFA centre of mass		RFA centre of mass
*t*	Time	τ	Time

**Table 2:** TMS bike designs with $\chi_{H}=1$.

Bike	$\chi_{H}$	$\chi_{B}$	$\zeta_{H}$	$\zeta_{B}$	$\omega_{0}$	$\lambda_{s}$ (rad)	${\mathrm{Fr}}_{c}$	${\mathrm{Fr}}_{\mathrm{EP}}$
${\mathrm{EP}}_{4}$	1	$\sqrt{2}$	−0.2	−0.4	$-\frac{\sqrt{5}}{\sqrt[{4}]{2}}$	$\arctan\left(\frac{15}{4}-\frac{75}{32}\sqrt{2}\right)\;\;\;\;\;\;\;\;\;\;\;$	$\frac{\sqrt{30\sqrt{2}+120}}{8}$	$\frac{3\sqrt{110\sqrt{2}-120}}{8}$
${2\mathrm{EP}}_{2}$	1	$\sqrt{2}$	−0.2	−0.4	$-\frac{\sqrt{5}}{\sqrt[{4}]{2}}$	$\arctan\left(\frac{15}{4}-\frac{75}{32}\sqrt{2}\right)-0.05$	≈1.482682090	≈2.257421384
${\mathrm{CEP}}_{2}$	1	$\sqrt{2}$	−0.2	−0.4	$-\frac{\sqrt{5}}{\sqrt[{4}]{2}}$	$\arctan\left(\frac{15}{4}-\frac{75}{32}\sqrt{2}\right)+0.80$	≈3.934331969	≈4.103508160
